# Role of nurses and the nursing assistants in the implementation and monitoring of physical restraint in psychiatry

**DOI:** 10.1192/j.eurpsy.2022.2266

**Published:** 2022-09-01

**Authors:** O. Maatouk, A. Larnaout, B. Abassi, R. Lansari, N. Staali, W. Melki

**Affiliations:** 1 Razi Hospital, F Adult Psychiatry Department, Manouba, Tunisia; 2 Razi Hospital, Psychiatry D, Manouba, Tunisia

**Keywords:** psychiatry, nursing assistant, nurse, physical restraint

## Abstract

**Introduction:**

Physical restraint is a therapeutic procedure allowing to immobilize an agitated patient.Although it is an effective method especially in the states of psychomotor instability, its practice is not devoid of risks which imposes a codified technique with particular monitoring.

**Objectives:**

The aim of this work was to evaluate the knowledge of nurses and nursing assistants in the practice and monitoring of physical restraint and to establish a suitble protocol codifing it.

**Methods:**

Our study was a descriptive cross-sectional study based on a questionnaire grouping together a set of questions on general and professional characteristics, the decision of physical restraint, its prescription, its means, its monitoring, informing the patient and his relatives, physical restraint’s risks, the patient’s experience, the caregiver’s experience as well as the relationship between caregiver and patient. Our target population was composed of nurses and orderlies of the psychiatry department <<D>> of the Razi hospital in Manouba.

**Results:**

We collected 30 professinals.90% of them were women. 30% of our sample had less than five years of experience. Only 23.30% of caregivers had mental health training at the beginning of their professional career. 50% of them received training focused on physical restraint.83.30% reported using physical restraint for psychomotor agitation.56.6% ignored the psychological effects of the physical straint on patients. 73.3% of caregivers informed patients before straint.

**Conclusions:**

A physical restraint protocol, codifying the technique of implementation and monitoring parameters is needed in order to improve the relation patient-cargiver and ensure an optimal care .

**Disclosure:**

No significant relationships.

